# GFP-complementation assay to detect functional CPP and protein delivery into living cells

**DOI:** 10.1038/srep18329

**Published:** 2015-12-16

**Authors:** Nadia Milech, Brooke AC Longville, Paula T Cunningham, Marie N Scobie, Heique M Bogdawa, Scott Winslow, Mark Anastasas, Theresa Connor, Ferrer Ong, Shane R Stone, Maria Kerfoot, Tatjana Heinrich, Karen M Kroeger, Yew-Foon Tan, Katrin Hoffmann, Wayne R Thomas, Paul M Watt, Richard M Hopkins

**Affiliations:** 1 Telethon Kids Institute & Centre for Child Health Research, The University of Western Australia, Drug Discovery Group. West Perth, 6872, Australia; 2 Phylogica. Subiaco East, Western Australia, 6008, Australia

## Abstract

Efficient cargo uptake is essential for cell-penetrating peptide (CPP) therapeutics, which deliver widely diverse cargoes by exploiting natural cell processes to penetrate the cell’s membranes. Yet most current CPP activity assays are hampered by limitations in assessing uptake, including confounding effects of conjugated fluorophores or ligands, indirect read-outs requiring secondary processing, and difficulty in discriminating internalization from endosomally trapped cargo. Split-complementation Endosomal Escape (SEE) provides the first direct assay visualizing true cytoplasmic-delivery of proteins at biologically relevant concentrations. The SEE assay has minimal background, is amenable to high-throughput processes, and adaptable to different transient and stable cell lines. This split-GFP-based platform can be useful to study transduction mechanisms, cellular imaging, and characterizing novel CPPs as pharmaceutical delivery agents in the treatment of disease.

The challenge for intracellular biologic drug delivery is achieving sufficient cytosolic uptake. A common solution is conjugating drugs to cell penetrating peptides (CPPs). Yet while many CPPs have been described, few CPP-delivered cargoes have entered the clinic^1^ with trials primarily dominated by just one CPP derived from the HIV transactivator protein (TAT)^2^,^3^. One reason so few of these therapeutics have made it to the clinic might be that many CPPs are generally inefficient at intracellular delivery and can remain largely trapped within endosomes^4^,^5^. Therefore, cargo proteins must normally be delivered at high concentrations exceeding 10 mg/kg to achieve biological efficacy^6^,^7^,^8^,^9^, but such high CPP concentrations become increasingly associated with cellular toxicity^10^. Differentiating between internalization and endosomal entrapment at therapeutically relevant concentrations is critical for discovering CPPs useful for research and for therapeutics.

It is also critical to demonstrate that internalized peptide is available to mediate biological functions. Various methods have been described for detecting CPP uptake. But none can be reliably used for exclusive detection of cytosolic delivery, and hence biological availability, via a simple direct read-out. For example, assays using labeled CPPs can be affected by background signal from fluorescently-labeled CPPs trapped in endosomes, or from the labeling fluorophore^11^,^12^,^13^ or steroid^14^,^15^ itself over-enhancing membrane penetration and cytoplasmic uptake. Other options lack a direct signal readout, for example splice-correction or transcription-factor delivery assays that require processing of the signal or additional events for readout such as nuclear translocation and reporter-gene expression^14^,^16^,^17^,^18^,^19^. Alternative assays that rely on the redox potential of early endosomes to keep disulphide bonds oxidized^20^,^21^ find that premise may not always be the case, especially for artificial constructs^22^. All such factors complicate the interpretation of conventional CPP assays.

This highlights the need for a diagnostic assay that specifically discriminates between cytoplasmic delivery and endosomal entrapment, is independent of the functional or differentiation state of the target cells, and also has a direct and easily-visualized readout with minimal background and sufficient sensitivity to detect CPP internalization at concentrations below toxic dosages. Our Split-complementation Endosomal Escape (SEE) assay visualizes cytosolic internalization of CPPs by fluorescence and thereby meets this need. The SEE platform is amenable to high-throughput processes and adaptable to different cell types (both transient and stable), making it a robust and sensitive diagnostic assay that specifically distinguishes functional uptake and cargo internalization in living cells.

## Results

### Building a diagnostic assay to specifically detect cytoplasmic uptake

**Figure 1:**
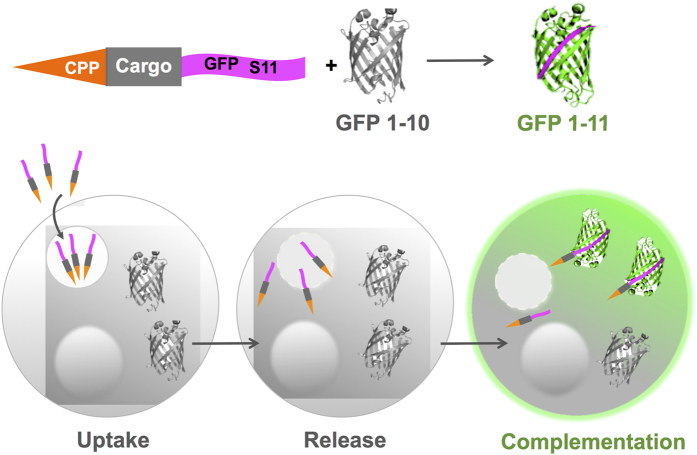
Split-complementation Endocytotic Escape (SEE): visualizing CPP functional uptake. Live-cell functional uptake is detected when a CPP-protein fused to S11-peptide penetrates the cell membrane to complement cytosolic GFP1-10 protein, forming a functional GFP fluorophore.

We chose the self-assembling split-GFP protein-solubility assay^23^,^24^ as a template in building a platform to specifically detect cytoplasmic uptake: the larger GFP1-10 protein fragment is expressed in the cytosol of cells while the GFP S11 protein fragment is fused to a CPP-containing moiety. Fusing CPPs to one part of a split assembly ensures that complementation only occurs if the fusion penetrates the cell membrane and enters the cytoplasm to form fluorescent GFP molecules (Fig. 1). CPPs and fusions trapped within endosomes cannot complement the cytoplasmic-expressed component and thus do not present false-positives or a strong background signal, making the SEE assay specific for detecting biologically available cargo delivery and for discriminating between CPPs based on function.

**Figure 2:**
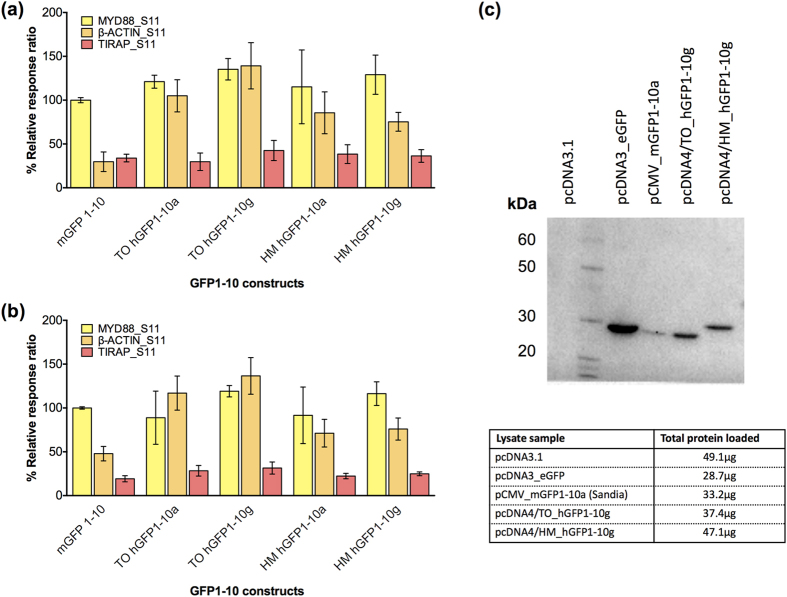
Optimization of GFP1-10 coding sequence and expression. (**2a,b)** Split-GFP complementation is observed as an increase in relative intensity of the fluorescence signal in HEK-293 cells co-transfected with different GFP1-10 moieties and complementing Cargo_S11 fusions and the resulting fluorescent signal was measured at 24 h (2a) and 48 h (2b) post transfection. Florescence is expressed as a relative ratio compared to the signal from murine-encoded GFP1-10 co-expressed with MYD88_S11 (set to 100%); error bars represent standard error of the mean between technical replicates. The highest complementation signal was seen using pcDNA4/TO-hGFP1-10 g (hGFP1-10), measured at both time points. Transfection constructs are detailed in Supplementary Table 1. (**2c**) Immunoblot of lysate from HEK-293 cells transfected with either: (i) control construct expressing no protein (empty vector control: pcDNA3.1 with minor modification to the MCS), (ii) a construct expressing eGFP (cloned in pcDNA3, immunoblot positive control), (iii) pCMV mGFP1-10a (Sandia, 26.6 kDa), (iv) pcDNA4/TO hGFP1-10 g expressing human-codon optimized GFP1-10 sequence from pcDNA4/TO (26.6 kDa), and (v) pcDNA4/HM hGFP1-10 g expressing human-codon optimized GFP1-10 sequence from pcDNA4/HM (30.1 kDa, hGFP1-10 is expressed with 3 N’ motifs provided by the vector (a His tag, Xpress tag and EK recognition cleavage site) which increase overall protein size). GFP and GFP1-10 expression is detected with an anti-GFP rabbit polyclonal primary antibody. Expression construct pcDNA4/TO hGFP1-10 g (hGFP1-10) shows the highest expression of GFP1-10 relative to total amount of lysate run on the SDS-PAGE gel.

Firstly we engineered the SEE platform components and improved the sensitivity and signal strength by optimizing our GFP1-10 fragment for human codon usage and by using different mammalian expression vectors to increase the amount of protein expressed within the cells (Supplementary Table 1). We analyzed complementation signals produced in co-transfections of HEK-293 cells with different GFP1-10 and Cargo_S11 constructs (Fig. 2a,b), and confirmed improved expression by comparing GFP1-10 expression levels (Fig. 2c), measuring GFP complementation with a fluorescence plate reader. The construct with strongest complementation signal and expression was human-codon optimized GFP1-10 expressed from pcDNA4/TO (this construct is hereafter referred to as hGFP1-10).

**Figure 3:**
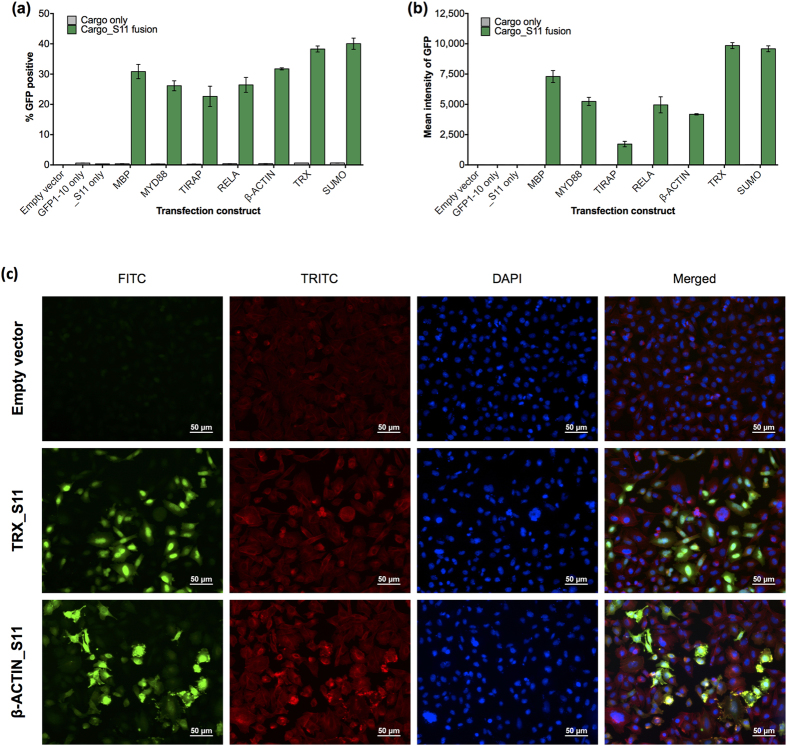
Validating SEE components by transfection. (**3a,b)** SEE components are validated by co-transfection of CHO-K1 cells with hGFP1-10 and various Cargo_S11 fusion constructs. All S11 fusions complemented hGFP1-10, measured by increasing % fluorescent cells (**3a**) or increasing MFI (**3b**) in the viable single-cell population. In all experiments the “S11 only” control is a fusion of the [GSSG]x2 linker and S11 sequence, and does not generate a detectable GFP signal, nor do empty vector and “GFP1-10 only” controls (% GFP-positive cells <0.65 or MFI <25). Error bars represent standard error of the mean between technical replicates; data is representative of three independent experiments. (**3c)** Fluorescence microscopy detects GFP complementation (FITC channel) in HCC827/hGFP1-10 cell populations transfected with complementing β-ACTIN_S11 or TRX_S11 constructs, compared to empty vector control. Cells are also counter-stained for endogenous β-Actin (TRITC) and nuclei (DAPI) before visualizing. Bar scale is 50 μm.

However the goal of SEE is to study CPP uptake in living cells. Therefore we elected to shift the platform analysis method to flow cytometry and include a cellular live/dead stain to enable exclusion of dead cell populations from the data analysis. Thus CHO-K1 cells were co-transfected with hGFP1-10 combined with various Cargo_S11 constructs, where the cargo proteins were linked to S11 by a [GSSG]x2 linker. The next day GFP1-10 complementation was measured by flow cytometry, analyzing the viable single-cell population for both signal strength (mean fluorescence intensity (MFI) of GFP) and percentage of fluorescing cells. All transfected Cargo_S11 fusions complemented hGFP1-10 and produced a fluorescent signal, while the S11 sequence alone did not (Fig. 3a,b). Fluorescence microscopy confirmed GFP signal only in cells co-transfected with complementing Cargo_S11 constructs (CHO-K1 cells, data not shown) or monoclonal cell lines stably expressing hGFP1-10 and transfected with S11-complementing constructs for different cargos (HCC827/hGFP1-10 cells, Fig. 3c); no signal was detected in empty vector control co-transfections. Taken together, these data validated the signal function of the SEE platform components in living cells.

### Optimizing SEE assay components to improve signal strength and detection

**Figure 4:**
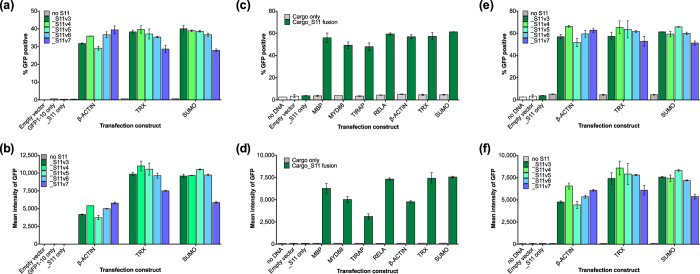
SEE component and linker validation in cells stably expressing hGFP1-10. (**4a,b)** CHO-K1 cells are co-transfected with hGFP1-10 and various Cargo-linker combinations. All_S11 fusions complemented hGFP1-10 to various extents, measured by increasing % fluorescent cells (**4a**) or increasing MFI (**4b**) in the viable single-cell population. In all experiments the “S11 only” control is a fusion of the [GSSG]x2 linker and S11 sequence, and does not generate a detectable GFP signal, nor do empty vector and “GFP1-10 only” controls (% GFP-positive cells <0.65 or MFI <25). Error bars represent standard error of the mean between technical replicates; data is representative of three independent experiments. (**4c–f)** Transfection experiments validating CPP components were repeated in a monoclonal stable cell line: HCC827 cells expressing hGFP1-10. HCC827/hGFP1-10 cells are transfected with different Cargo_S11 fusion constructs (**4c,d**) or various Cargo-linker combinations (**4e,f**). All Cargo_S11 proteins and various Cargo-linker combinations complemented hGFP1-10, as measured by increasing % GFP-positive cells (**4c,e**) or increasing MFI (**4d,f**) in the viable single-cell population and compared to background (no GFP1-10 and/or empty vector). In all experiments the “S11 only” control is expressed [GSSG]x2 linker_S11 fusion and does not generate a detectable increase in GFP signal, nor do empty vector and “GFP1-10 only” controls (% GFP-positive cells <5%, or MFI <110). Error bars represent standard error of the mean between technical replicates; data is representative of three independent experiments.

We fine-tuned the platform by optimizing the linker that separates protein cargoes and S11. This linker provides a spacer distance between the cargo and the S11 fragment and ensures adequate freedom-of-movement for the S11 fragment to fold with and complement GFP1-10 and minimizing any potential steric hindrance. Linker variants (linkers 4–7) were designed by analyzing component domain structures of three cargos: a cellular protein (β-ACTIN) and two solubility enhancers of recombinant protein expression (SUMO and Thioredoxin (TRX)), both of which showed strong and widespread complementation signals compared to the more localized expression of β-ACTIN_S11 within the cell (Fig. 3c and data not shown). Linker effects were evaluated by co-transfecting the linker-variant Cargo_S11 fusions with hGFP1-10 and GFP complementation was again detected by flow cytometry, analyzing the viable single-cell population for both signal strength and percentage of fluorescing cells. Overall, the different linker variants gave a small increase to the assay signal (Fig. 4a,b). For final component validation we repeated these experiments in our monoclonal HCC827 cells that stably expressed hGFP1-10, transfecting S11-complementing constructs for different cargos (Fig. 4c,d) and linker variants into the cells (Fig. 4e,f).

GFP complementation patterns were comparable across different cargos and cargo-linker combinations in the stable cell line compared to hGFP1-10 transiently-transfected cells, verifying that all SEE components function with transient or stable expression of GFP1-10. One key advantage of using hGFP1-10 stable cell lines is that it minimizes potential signal differences due to variability in hGFP1-10 transfection efficiency. The low endogenous background signal in stable cells from the marginal fluorescent capability of hGFP1-10 is easily filtered by appropriately placed gates in flow cytometry analysis and is dwarfed by true GFP complementation. For platform validation we chose TRX as the optimal cargo for further studies as it had a strong detectable signal in co-transfection experiments and has been regularly used in our laboratory’s recombinant expression work. Linker 4 ([GSSG]x4) was chosen as the preferred linker for its length, flexibility and retention of strong GFP complementation signal with TRX.

### SEE specifically detects CPP-dependent delivery into the cytoplasm of cells

The ideal CPP assay adds proteins to cells to produce a directly measured readout that differentiates functionally internalized protein. To test SEE by this standard we expressed recombinant proteins displaying: (i) an N-terminal CPP, (ii) TRX, and (iii) a C-terminal S11 sequence (Supplementary Table 2). Each of these elements was separated by a short, flexible linker that includes unique restriction sites in the encoding DNA for rapid element-shuttling, while an internal His tag was incorporated for protein purification and proved extremely successful. A range of canonical CPPs were selected, representing different CPP classes^25^,^26^: TAT^27^, R9^28^, Penetratin^29^ (PEN), Penetratin-Arginine^30^ (PenArg), SAP^31^, VP22^32^, PEP1^33^, hCT^34^, PTD4^35^, Ypep^36^, and Transportan^37^. In parallel we produced control proteins without an N-terminal CPP (TRX_S11) or where the “CPP” was a short peptide sequence with no cell-penetrating activity (PYC35^38^,^39^, fusion protein PYC35_TRX_S11).

**Figure 5:**
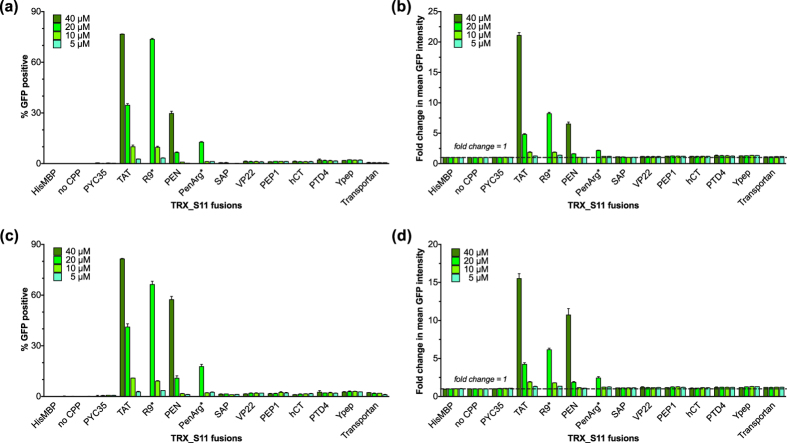
Recombinant CPP Cargo_S11 internalization is specifically detected in live cells. CPP_TRX_S11 proteins were added to CHO-K1 (**5a,b**) and HCC827 (**5c,d**) monoclonal cell lines stably expressing hGFP1-10. Protein titration shows dose-dependent uptake for TAT, Penetratin (PEN), Penetratin-Arginine (PenArg) and R9 fusions read as GFP complementation measured by both % fluorescent cells (**5a,c**) and fold change in MFI (**5b,d**). The seven other canonical CPPs showed only minimal GFP complementation signal at the highest doses. Control TRX_S11 fusion proteins “No CPP” and “PYC35” (a peptide with no CPP activity) show negligible effect on GFP complementation signal over cell-line background (“HisMBP”). Error bars represent standard error of the mean between technical replicates; data is representative of two independent experiments per cell line. (*R9 and PenArg fusions were not sufficiently concentrated for testing at 40 μM.)

Proteins were incubated on HCC827 and CHO-K1 cells stably expressing hGFP1-10 and complementation was detected the following day. Control proteins produced negligible fluorescent signals above background, establishing that there is no uptake potential inherent in the TRX_S11 fusion itself. In sharp contrast, a variety of CPP_TRX_S11 proteins greatly increased the signal, indicating functional cytoplasmic delivery. In particular, TAT, PEN, PenArg and R9_TRX_S11 proteins produced a dose-dependent complementation signal when measuring either percentage of fluorescing cells (Fig. 5a,c) or the fold-change in MFI (Fig. 5b,d). Contrary to expectations, none of the seven other conventional CPPs assayed showed significant cytoplasmic delivery, even at concentrations higher than physiologically desirable for drug delivery (40 μM).

**Figure 6:**
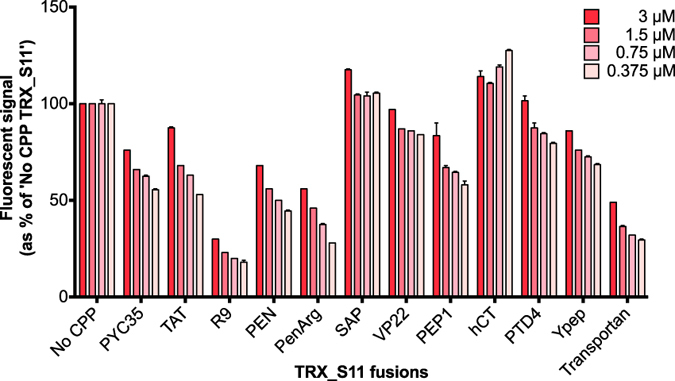
*In vitro* split-GFP protein complementation assay. CPP_TRX_S11 recombinant proteins (3 – 0.375 μM) are mixed *in vitro* with *E. coli*-expressed GFP1-10 (12.5 μM) and the resulting fluorescent signal read on a microplate reader. All CPP_TRX_S11 proteins show functional GFP complementation (presented as a percentage of the concentration-matched ”No CPP”_TRX_S11 sample), and a 10-fold decrease in S11 fusion protein concentration is neither sufficient to ablate the fluorescent signal, nor to decrease it by 10-fold. Error bars represent standard error of the mean between technical replicates.

**Figure 7:**
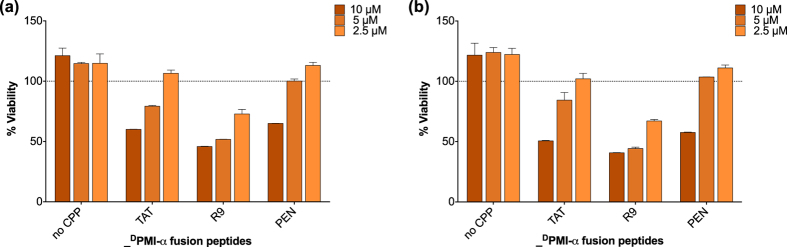
Recapitulation of CPP functional activity and ranking in an independent cell toxicity assay. Synthetic peptides are added to T47D cells and incubated for 24 hours in a DPMI-α cell toxicity assay, where peptide uptake results in a decrease in cell viability. Dose-dependent peptide toxicity is only detected for CPP DPMI-α fusions at both 24 h (**7a**) and 48 h (**7b**), illustrating CPP-dependent internalization. Here, “No CPP” control is the DPMI-α peptide without a CPP. Data is representative of two independent experiments. Error bars represent standard error of the mean between technical replicates.

We established the lack of complementation signal in living cells was not due to an inability of the CPP proteins to complement hGFP1-10 by mixing these same S11 fusions with recombinant soluble GFP1-10 *in vitro* to prove their ability to complement. All produced a dose-responsive fluorescent signal (Fig. 6) stronger than the basal signal of GFP1-10 or S11 alone, while a positive signal in the SEE assay does not correlate with high *in vitro* complementation signal. To independently validate the SEE assay’s ability to detect functional CPP activity, we assessed the bioactivity of SEE-positive CPPs in a cell toxicity assay where the CPPs were conjugated with the DPMI-α peptide^40^, which is cytosolic-toxic in cells highly-expressing *MDM2*. Dose-dependent toxicity was observed only for CPP delivery of the peptide cargo, at both 24 and 48 h (Fig. 7a,b); cargo alone was not toxic. Thus functional uptake of the CPP peptides is confirmed in both assays with their different cargoes, and relative CPP strength is consistent. We conclude that SEE can functionally differentiate between the CPPs based on biologically available internalized cargo and those unable to show cytosolic uptake.

**Figure 8:**
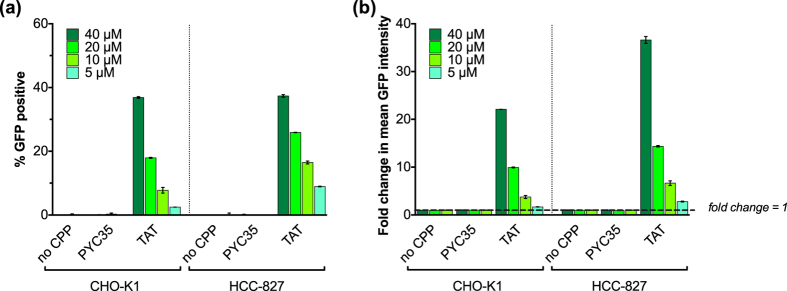
SEE CPP activity: recombinant CPP Cargo_S11 internalization is also detected in live cells transiently-transfected with GFP1-10. Recombinant proteins are added to CHO-K1 and HCC827 cells transiently transfected 24-hours prior with hGFP1-10 and left overnight to recover and express the protein. GFP complementation was measured as both % fluorescent cells (**8a**) and fold change in MFI (**8b**). A dose-dependent fluorescent signal is detected for the TAT fusion protein compared to controls illustrating CPP-dependent internalization in transfected cells. “No CPP” control is the TRX_S11 protein with no CPP moiety added. “PYC35” control is an unrelated peptide (known to have no CPP activity) instead of a CPP moiety expressed as part of the fusion protein. Data is representative of more than 10 independent experiments. Error bars represent standard error of the mean between technical replicates.

To establish SEE’s versatility, we repeated a SEE assay using TAT in cells transiently transfected with hGFP1-10 the day before protein addition: CPP-driven intracellular uptake was clearly distinguishable over control proteins down to the 5 μM protein dose (Fig. 8a,b). The MFI signal was greater compared to SEE in stable cell lines, likely due to increased copies of hGFP1-10 within individual cells. These data demonstrate that the SEE platform is flexible and adaptable to cell lines of choice, depending on whether transient or stable transfection of GFP1-10 is preferred. Moreover, the assay time-frame for protein folding is compatible with both fast and slow uptake CPPs (e.g., TAT and PEN, respectively^21^).

## Discussion

The SEE assay addresses a major challenge for expanding the toolbox of protein therapeutics – identifying effective peptide sequences capable of penetrating cell membranes. Such cell-penetrating peptides find numerous uses in targeting intercellular proteins and carrying diverse cargos into cells, including peptides, small molecules, DNA, RNA and proteins. CPP-carried therapeutics can be administered in various ways from injection to gel-based formulations that offer a topical administration alternative, and they open up the therapeutic space for intracellular drug targets, largely a still-untapped area. The challenge in intracellular biologic drug research is identifying those CPPs that achieve good cytosolic uptake from those that don’t.

SEE is robust and sensitive over a dynamic range and is independent of the mechanism of internalization, as shown by detection of: 1) even small amounts of complementation despite decreased protein concentrations that result in less molecules of complemented GFP per cell; and 2) increased complementation signals at high protein concentrations where cationic peptides can internalize non-specifically from non-specific “flooding” via sphingomyelin-to-ceremide conversion^41^. Moreover incorporating GFP variations with higher quantum efficiency^42^ or faster reassembly times^43^ offers the future potential for greater sensitivity in detecting even small amounts of complemented fluorophore or discriminating slow- from fast-uptake CPPs by measuring complementation signal at earlier time points. When used to compare a panel of conventional CPPs, SEE revealed striking differences between their abilities to internalize a cargo protein as visualized through split-GFP complementation, thus discriminating between well-characterized CPPs based on functional uptake efficiency. As CPP function can be affected by the cargo attached, sometimes relative ranking of CPP strength is also influenced by the cargo. However, at pharmacologically relevant concentrations (10 μM or less), TAT and R9 showed the best cytoplasmic uptake of the CPPs tested in the SEE assay. The relative strength of these conventional CPPs showing unenhanced functional uptake in both the SEE and an independent cell toxicity assay is also consistent with previous rankings facilitated by an uptake enhancer^44^.

SEE is a novel and simple functional assay that reliably distinguishes between intracellular delivery and cargo not biologically available, regardless of method of internalization (e.g., receptor-mediated, macropinocytosis, etc.). Its advantages include minimal background, dynamic range, direct readout that is independent of signaling pathways or enzymatic processing, compatibility with high throughput processes, and applicability to differing cell lines. And while the assay was used here to assess functional cytoplasmic uptake of CPPs, it could be used to measure internalization of any uptake molecule conjugated to the complementation component. Combining SEE with endosomolytic agents such as bifurcated TAT^45^ offers the potential to quantify functional CPP uptake, a key question for pharmacological delivery of bio-therapeutics. The SEE principle itself is also easily adaptable to other split complementation systems, for example split-luciferase or other fluorescent proteins of different spectrums (e.g., red, yellow, etc.). And we speculate SEE transgenic mice may offer an environment for *in vivo* imaging of intracellular and/or site-specific drug delivery. Finally, the power of SEE comes not only in its immediate application of evaluating and discriminating between functional cell-penetrating activity of CPPs or cell-specific ligands, but also in the opportunity to combine SEE assessment with internalization inhibitors to facilitate mechanism studies of action and cell specificity in the pharmacological development of CPPs as delivery agents in the treatment of disease.

## Methods

### Plasmids

DNA sequences for β-ACTIN (NP 001092.1), MYD88 (NP 002459.2), TIRAP (AF406652 1), RELA (NP 068810.3) and TRX (EDV64981), SUMO^46^ and split-GFP components^23^,^24^ were synthesized (DNA2.0) and codon-optimized for human or *E. coli* codon usage for mammalian or recombinant protein expression constructs, respectively (see Supplementary Table 1). Plasmid stocks were prepared using Plasmid Plus DNA kits (QIAGEN).

### Modeling of Cargo_S11 linkers

Crystal structures for cargo proteins (TRX (1AIU)^47^, β-ACTIN (2OAN)^48^, SUMO (2UYZ)^49^) and complemented S11/split-GFP were evaluated using PyMOL (PyMOL Molecular Graphics System, Version 1.30, Schrödinger, LLC). GFP crystal-structure (1KP5)^50^ analysis indicated the core β-barrel span (excluding loops) was approximately 22 Å, and the full span (including loops) 42 Å. These distances were the separation criteria between the cargoes and S11, so as to keep them approximately one GFP domain distance apart; a set of 5 flexible linkers between 8–18 amino acids long were designed to cover the 22–42 Å space. **Linker sequences:** Linker 3 (v3): [GSSG]x2; Linker 4 (v4): [GSSG]x4; Linker 5 (v5): [GGTGGSGGA]x2; Linker 6 (v6): GSSGGSSGGGTGGS; Linker 7 (v7): [AP]x5.

### Synthetic peptides

Peptides were synthesized (Pepscan) to >90% purity. CPPs are separated from the DPMI-α peptide by a GAS linker. **Peptide sequences:** DPMI-α: tnwyanlekllr; TAT DPMI-α: GRKKRRQRRRGAStnwyanlekllr; R9 DPMI-α: RRRRRRRRRGASt-nwyanlekllr; PEN DPMI-α: RQIKIWFQNRRMKWKKGAStnwyanlekllr.

### Mammalian cell culture

CHO-K1, HCC827, and T47D cells (ATCC, verified mycoplasma-free) were cultured in RPMI with 10% FCS, 4 mM Glutamax (LifeTech), 100 U/ml penicillin, and 100 ug/ml streptomycin. HCC827 was additionally supplemented with 10 mM HEPES, 1x nonessential amino acids and 1 mM sodium pyruvate. HEK-293 cells were cultured in DMEM supplemented with 10 % FCS, 4 mM Glutamax, 100 U/ml penicillin, and 100 ug/ml streptomycin. All cells were cultured at 37 ˚C under 5 % CO_2_. CHO-K1 and HCC827 monoclonal cell lines stably expressing hGFP1-10 were made through limiting dilution by Genecopoeia (USA), and were maintained in Zeocin-supplemented parental cell line medium with 200 ug/ml and 250 ug/ml Zeocin, respectively. For split-GFP transfection and recombinant protein experiments, cells were grown in complete medium lacking antibiotics. Unless otherwise stated, adherent cells were seeded for assays in 96-well flat-bottomed clear polystyrene plates and suspension cells grown in similar round-bottomed plates; cells were transfected at 60–80 % confluence using Lipofectamine LTX Plus (Invitrogen).

### SDS-PAGE and Immunoblotting

HEK-293 cells were transiently transfected with GFP1-10 constructs. After 48 hours cells were washed twice with PBS and lysed with M-PER Lysis buffer (Pierce) containing 1x cOmplete Ultra protease inhibitors (Roche). Equal volumes of lysate were separated by SDS-PAGE (4–12 % BisTris NUPAGE gel, Invitrogen) and transferred to PVDF membrane by iBlot (Invitrogen). Immunoblots were processed in the BenchPro blotting system (Invitrogen), using an anti-GFP rabbit primary antibody (G1544, Sigma) and anti-rabbit IgG-HRP secondary antibody (NA9340V, Amersham). Blots were visualized with ECL reagents (Amersham) and images captured by ChemiDoc Imager (BioRad).

### Split-GFP transfection assay

HEK-293, CHO-K1 or HCC827/hGFP1-10 cells were seeded (10,000, 10,000 or 7,500 cells/well, respectively) into 96-well plates pre-coated with gelatin and co-transfected the next day with equal amounts of either Cargo_S11 or_S11 linker variant constructs and hGFP1-10 (HEK-293 and CHO-K1) or pcDNA3.1+ (HCC827/hGFP1-10). In initial experiments fluorescence was assayed 24 h and 48 h post-transfection using a Synergy Mx plate reader (BioTek Instruments, Inc.) with 488 nm/9 and 525 nm/9 excitation/emission filters. Data was normalized to mGFP1-10 co-transfected with MYD88_S11 (set to 100%) to produce the Relative Response Ratio. Error bars represent standard error of the mean between duplicate samples. Otherwise, after 24 h cells were prepared for flow cytometry.

### SEE CPP assay

Cells were transfected with hGFP1-10 with Neon Transfection (Invitrogen) and seeded into 96-well plates at 20,000 (CHO-K1) and 30,000 cells/well (HCC827). Stable cell lines were seeded at 10,000 cells/well. The next day recombinant CPP_TRX_S11 proteins were added to final concentrations of 5, 10, 20 or 40 μM. After 24 h, cells were prepared for flow cytometry.

### Flow cytometry

Cells were detached from plates with 0.25 % trypsin/EDTA (Gibco), washed and stained with Violet Live/Dead stain (Molecular Probes) then processed on a LSRFortessa flow cytometer with FACS DIVA software (BD Biosciences), excited with a 488 nm blue laser and selected with a 530/30 filter. The Violet Live/Dead stain was used for the measurement of dead cells detected by excitation with a 405 nm violet laser and selection by 450/50 filters. Data were analyzed with FlowJo X 10.0.7 software (Tree Star Inc.). Gating of the single cell population (distributed FSC-H vs. FSC-W) was followed by gating of the viable single cell population according to live/dead stain status, and this viable single cell population then further divided into green (FITC+) and not green (FITC-). For analysis, samples were corrected for background by subtracting the “No CPP”_TRX_S11 data for the matched dose. To calculate fold change of MFI, sample data was divided by the corresponding dose “No CPP”_TRX_S11 (thus all “No CPP”_TRX_S11 samples become fold change of 1). For Fig. 8 only, where TRX _S11 control was not available at 5 μM, the next highest dose control (10 μM) was subtracted from the 5 μM test samples. Error bars represent standard error of the mean between duplicate samples.

### Fluorescent microscopy

HCC827/hGFP1-10 cells (25,000 cells/well) were grown overnight in LabTek II CC2 glass chamber slides (Nalge Nunc International) then transfected with DNA (400 ng/well). After 24 h cells were fixed, permeabilized and blocked using the Image-iT Fixation/Permeabilization Kit (Molecular Probes). After blocking, cells were stained for β-Actin and nuclei using ActinRed and NucBlue staining kits (Molecular Probes), washed 3 times with PBS and mounted with ProlongDiamond Antifade (Molecular Probes). Fluorescence images were acquired on a Nikon C2 plus microscope (standard FITC, TRITC and UV fluorescence filters) through a 20x DIC objective and using NIS Elements software. Exposure times for each channel were kept constant between each sample: Nuclei (UV) 600 ms, GFP (FITC) 300 ms, ActinRed (TRITC) 200 ms.

### Cell viability assay

T47D cells were cultured in phenol red-free RPMI supplemented with 2 % FCS, 100 U/mL penicillin and 100 ug/ml streptomycin. Cells were seeded at 8,000 cells/well in 96-well plates, incubated for 24 h, and then treated with the DPMI-α peptides at indicated doses. After 24 and 48 h PrestoBlue® reagent (LifeTech) was added to the media according to manufacturer’s instructions. Cells were incubated for a further 30 min and fluorescence read using an EnSpire® multimode plate reader (Perkin Elmer). Error bars represent standard error of the mean between duplicate samples.

### Recombinant protein expression

Recombinantly-expressed proteins display: (i) N-terminal CPP, (ii) TRX protein cargo, and (iii) C-terminal S11 sequence. Each moiety is separated by a short linker sequence that supplies unique restriction sites in coding DNA to facilitate rapid shuttling of cargo and CPP combinations. An internal His tag is present for protein purification. Proteins were expressed in BL21(DE3)-Gold cells (Agilent Technologies), except pET28 SGFP S1-10 that was expressed in Origami2(DE3) cells (Merck Millipore). Bacteria were cultured at 30 °C, 250 rpm for 24 h (500 ml Overnight Express™ Instant TB Medium, Merck Millipore). Cells were collected by centrifugation (5,000 rpm, 20 min, 4 °C), washed with 50 ml PBS (pH7.4), and resuspended in 30 ml IMAC binding buffer (20 mM phosphate, 500 mM NaCl, 20 mM imidazole, pH 8.0) before sonication (three pulses of 1 min).

### Recombinant protein purification

HisMBP and S11 proteins were purified using the FPLC AKTÄxpress system (GE Healthcare) at room temperature (RT). Briefly, lysates were clarified by centrifugation (20,000 rpm, 30 min, 4 °C) and soluble fractions purified on HisTrap HP 5 ml columns (GE Healthcare) pre-equilibrated with IMAC binding buffer and eluted with a linear gradient of IMAC elution buffer (20 mM phosphate, 500 mM NaCl, 500 mM imidazole, pH 8.0) to 100% at 5 ml/min flow rate. Fractions containing homogeneous S11 protein were pooled, concentrated (Amicon Ultra-15, MWCO 10 K; Merck Millipore) and buffer-exchanged with PBS (pH7.4) using PD-10 desalting columns (GE Healthcare). Average recombinant protein purity was 85 % by densitometry assessment (ChemiDoc MP System, BioRad) on Gel Code Blue Reagent-stained 4–20 % SDS-PAGE gels (Biorad).

Split-GFP 1–10 (SGFP S1-10) was purified with IMAC gravity flow protocol at RT. Briefly, lysate was clarified by centrifugation (20,000 rpm, 30 min, 4 °C) and the soluble fraction purified on Econo-Pac chromatographic column (BioRad) containing 2 ml 50 % Ni Sepharose High Performance slurry (GE Healthcare) pre-equilibrated with IMAC binding buffer, mixing by rotation for 10 mins to allow binding to resin. Columns were washed with binding buffer (4X column volumes), protein eluted with 2.5 ml IMAC elution buffer, and this eluate dialyzed against TNG buffer (50 mM Tris pH7.4, 0.15 mM NaCl, 10% Glycerol), using a PD-10 desalting column (GE Healthcare). Protein purity was confirmed by analysis on 4–16 % SDS-PAGE stained with Gel Code Blue Reagent.

### Split-GFP *in vitro* complementation assay

Microplates were blocked with gentle shaking for 10 mins at RT with 100 ul 0.5 % (w/v) bovine serum albumin (BSA) in TNG buffer. Recombinant S11 proteins were serial diluted in TNG from 3 μM to 0.375 μM, and then 20 ul added to the plate in duplicate. For complementation, 180 ul of split GFP S1-10 (12.5 μM in TNG buffer) was added and mixed. Negative controls contained only 0.5 % (w/v) BSA in TNG buffer mixed with recombinant split GFP S1-10. Samples were incubated overnight at RT and fluorescence (excitation wavelength = 488 nm; emission wavelength = 530 nm; 25 flashes) measured with an EnSpire Multimode Plate Reader (PerkinElmer). In analysis, raw data was corrected for background (subtracting TNG buffer and split GFP S1-10 controls). Sample data is presented as a percentage of the concentration-matched ”No CPP” control (set to 100 %).

## Additional Information

**Accession codes**: Accession codes referenced: β-ACTIN (NP 001092.1), MYD88 (NP 002459.2), TIRAP (AF406652 1), RELA (NP 068810.3) and TRX (EDV64981).

**How to cite this article**: Milech, N. *et al.* GFP-complementation assay to detect functional CPP and protein delivery into living cells. *Sci. Rep.* **5**, 18329; doi: 10.1038/srep18329 (2015).

## Supplementary Material

Supplementary tables
