# Supplementary material for: GFP-complementation assay to detect functional CPP and protein delivery into living cells
Source: Sci Rep. 2015 Dec 16;5:18329. doi: 10.1038/srep18329 (PMC4680871; doi:10.1038/srep18329)
Supplement: Supplementary tables [file srep18329-s1.pdf]

SUPPLEMENTARY INFORMATION

Manuscript:

GFP-complementation assay to detect functional CPP and protein delivery into living cells

Nadia Milech[1,*], Brooke AC Longville[1], Paula T Cunningham[1], Marie N Scobie[1], Heique M Bogdawa[1], Scott Winslow[1], Mark Anastasas[1], Theresa Connor[1], Ferrer Ong[1], Shane R Stone[1], Maria Kerfoot[1], Tatjana Heinrich[1], Karen M Kroeger[2], Yew-Foon Tan[1], Katrin Hoffmann[1], Wayne R Thomas[1], Paul M Watt[2,+], and Richard M Hopkins[2,+]

[1]Telethon Kids Institute & Centre for Child Health Research, The University of Western Australia, Drug Discovery Group. West Perth, 6872, Australia
[2]Phylogica. Subiaco East, Western Australia, 6008, Australia

[*] Nadia.Milech@telethonkids.org.au

[+] These authors contributed equally to this work.

SUPPLEMENTARY TABLES

Supplementary Table 1: Genetic constructs for mammalian experiments

Plasmid vector	Construct variants	Notes
pcDNA3 (Invitrogen)	eGFP	Used as control for high GFP expression in immunoblotting
pcDNA3.1 (Invitrogen)		Empty vector used for transfection control
	MBP	Human codon-optimised MBP sequence; negative control for MBP_S11
	MYD88	MYD88 sequence (NP_002459.2), negative complementation control for MYD88_S11
	TIRAP	TIRAP sequence (AF406652_1), negative complementation control for TIRAP_S11
	RELA	RELA sequence (NP_068810.3), negative complementation control for RELA_S11
	β-ACTIN	β-ACTIN sequence (NP_001092.1), negative complementation control for β- ACTIN_S11
	TRX	Thioredoxin (TRX) sequence (EDV64981), negative complementation control for TRX_S11
	SUMO	SUMO sequence variant where alanine-arginine replaces the final diglycine motif,[1] negative complementation control for SUMO_S11
pCMV_mGFPS11 (Sandia)	_S11 (or “S11 only”)	Expresses fusion peptide: linker (v3), S11 sequence
	MBP_S11	Expresses fusion protein: MBP cargo, linker (v3), S11
	MYD88_S11	Expresses fusion protein: MYD88 cargo, linker (v3), S11
	TIRAP_S11	Expresses fusion protein: TIRAP cargo, linker (v3), S11
	RELA_S11	Expresses fusion protein: RELA cargo, linker (v3), S11
	β-ACTIN_S11	Expresses fusion protein: β-ACTIN cargo, linker (v3, v4, v5, v6, or v7), S11
	TRX_S11	Expresses fusion protein: TRX cargo, linker (v3, v4, v5, v6, or v7), S11
	SUMO_S11	Expresses fusion protein: SUMO cargo, linker (v3, v4, v5, v6, or v7), S11
pCMV_mGFP1-10 (Sandia)		Murine codon-optimized GFP1-10; contains a single base mutation in the coding sequence (g466a) resulting in an amino acid change (N156D) from eGFP (U55762.1)
pcDNA4/TO (Invitrogen)	_hGFP1-10g	Human codon-optimized; GFP1-10 amino acid sequence has 100% identity compared to eGFP (U55762.1)
	_hGFP1-10a	Human codon-optimized; GFP1-10 amino acid sequence has a single base mutation (g466a) resulting in an amino acid change (N156D)
pcDNA4/HM (Invitrogen)	_hGFP1-10g	Human codon-optimized; amino acid sequence is the same as pcDNA4/TO_hGFP1-10g; hGFP1-10 is expressed with 3 N’ motifs provided by the vector (a His tag, Xpress tag and EK recognition cleavage site) which increase overall protein size
	_hGFP1-10a	Human codon-optimized; amino acid sequence is the same as pcDNA4/TO_hGFP1-10a; hGFP1-10 is expressed with 3 N’ motifs provided by the vector (a His tag, Xpress tag and EK recognition cleavage site) which increase overall protein size

Supplementary Table 2: Recombinant proteins used in this study

CPP sequences (reviewed in[2,3], also[4]) were synthesized as a gene fusion 5’ to the TRX and S11 coding sequences using *E. coli*-optimized codons (DNA2.0) and cloned into pET28a+ (Novagen). HisMBP was expressed from the pDestHisMBP plasmid. TRX functions as a solubility enhancing protein, which can be particularly effective when using CPPs such as TAT that may precipitate at high concentrations.[5]

Protein expressed	N’ sequence (eg, CPP)	His linker	Protein cargo (His-TRX)	Linker 4	S11	KDa	pI
_TRX_S11	none	GGTSHHHHHH	MSDKIIHLTDDSFDTDVL KADGAILVDFWAEWCGPC KMIAPILDEIADEYQGKL TVAKLNIDQNPGTAPKYG IRGIPTLLLFKNGEVAAT KVGALSKGQLKEFLDANL A	[GSSG] x4	GRDHMVLHEY VNAAGIT	16.4	5.77
PYC35_TRX_S11	GAYQSIRSGGIESSSKRER					18.5	6.1
TAT_TRX_S11	GRKKRRQRRR					17.8	8.6
R9_TRX_S11	GRRRRRRRRR					17.9	8.9
PEN_TRX_S11	GRQIKIWFQNRRMKWKK					18.7	7.8
PenArg_TRX_S11	GRQIRIWFQNRRMRWRR					18.8	7.8
Transportan_TRX_S11	GWTLNSAGYLLGKINLKALAALAKKIL					19.8	6.5
SAP_TRX_S11	GVRLPPPVRLPPPVRLPPP					18.5	6.3
VP22_TRX_S11	GDAATATRGRSAASRPTERPRAPARSASRPRR PVD					20.1	7.1
PEP1_TRX_S11	GKETWWETWWTEWSQPKKKRKV					19.3	6.3
hCT_TRX_S11	GLGTYTQDFNKTFPQTAIGVGAP					18.8	5.8
PTD4_TRX_S11	GYARAAARQARA					17.68	6.3
Ypep_TRX_S11	GYTFGLKTSFNVQ					17.9	5.9

Protein expressed	Protein sequence	KDa	pI
GFP1-10	MSKGEELFTGVVPILVELDGDVNGHKFSVRGEGEGDATIGKLTLKFICTTGKLPVPWPTLVTTLTYGVQCFSRYPDHMKRHDFFKS AMPEGYVQERTISFKDDGKYKTRAVVKFEGDTLVNRIELKGTDFKEDGNILGHKLEYNFNSHNVYITADKQKNGIKANFTVRHNVE DGSVQLADHYQQNTPIGDGPVLLPDNHYLSTQTVLSKDPNEK	26.6	6.51
HisMBP *(Control added to stable cell lines to mark baseline fluorescence)*	MKIHHHHHHEEGKLVIWINGDKGYNGLAEVGKKFEKDTGIKVTVEHPDKLEEKFPQVAATGDGPDIIFWAHDRFGGYAQSGLLAEI TPDKAFQDKLYPFTWDAVRYNGKLIAYPIAVEALSLIYNKDLLPNPPKTWEEIPALDKELKAKGKSALMFNLQEPYFTWPLIAADG GYAFKYENGKYDIKDVGVDNAGAKAGLTFLVDLIKNKHMNADTDYSIAEAAFNKGETAMTINGPWAWSNIDTSKVNYGVTVLPTFK GQPSKPFVGVLSAGINAASPNKELAKEFLENYLLTDEGLEAVNKDKPLGAVALKSYEEELAKDPRIAATMENAQKGEIMPNIPQMS AFWYAVRTAVINAASGRQTVDEALKDAQTNS	41.36	5.48

1. Vitte, A.-L. & Jalinot, P. Intracellular delivery of peptides via association with ubiquitin or SUMO-1 coupled to protein transduction domains. *BMC Biotechnol.* **8,** 24 (2008).
2. Heitz, F., Morris, M. C. & Divita, G. Twenty years of cell-penetrating peptides: from molecular mechanisms to therapeutics. *Br J Pharmacol* **157,** 195–206 (2009).
3. Jones, A. T. & Sayers, E. J. Cell entry of cell penetrating peptides: tales of tails wagging dogs. *Journal of Controlled Release* **161,** 582–591 (2012).
4. Åmand, H. L., Fant, K., Nordén, B. & Esbjörner, E. K. Stimulated endocytosis in penetratin uptake: effect of arginine and lysine. *Biochem Biophys Res Commun* **371,** 621–625 (2008).
5. Becker-Hapak, M., McAllister, S. S. & Dowdy, S. F. TAT-mediated protein transduction into mammalian cells. *Methods* **24,** 247–256 (2001).
